# Recent advances in treating Parkinson’s disease

**DOI:** 10.12688/f1000research.10100.1

**Published:** 2017-03-13

**Authors:** Wolfgang H. Oertel

**Affiliations:** 1 Department of Neurology, University Clinic, Philipps Universität Marburg, Marburg, Germany; 2 Institute for Neurogenomics, Helmholtz Center for Health and Environment, Munich, Germany

**Keywords:** Parkinson's disease, prodromal stage, motor symptoms, non-motor symptoms, disease modifying treatment

## Abstract

This article summarizes (1) the recent achievements to further improve symptomatic therapy of motor Parkinson’s disease (PD) symptoms, (2) the still-few attempts to systematically search for symptomatic therapy of non-motor symptoms in PD, and (3) the advances in the development and clinical testing of compounds which promise to offer disease modification in already-manifest PD. However, prevention (that is, slowing or stopping PD in a prodromal stage) is still a dream and one reason for this is that we have no consensus on primary endpoints for clinical trials which reflect the progression in prodromal stages of PD, such as in rapid eye movement sleep behavior disorder (RBD) —a methodological challenge to be met in the future.

## Introduction

### The decades of focus on the nigrostriatal system and dopamine replacement therapy

Parkinson’s disease (PD) is a devastating disorder of the human nervous system and the second most common progressive chronic neurodegenerative disease. The three cardinal motor symptoms, akinesia in combination with either tremor at rest or rigidity ( 1 UK brain bank criteria), are—200 years after their description—still the basis of the clinical diagnosis. Up to 2016, we still have no treatment to slow down or even stop the progression of the disease. Available therapy is symptomatic. This article presents evidence that for the first time in history substances with a potentially disease-modifying effect for PD are under development and thus offer hope for the patient, the spouse, and the treating physician.

For more than 100 years, we know the neuropathological hallmarks of the disorder: the so-called Lewy bodies ^2^ (proteinaceous intracytoplasmic inclusion bodies) containing aggregations of the protein alpha-synuclein ^3^ and the loss of pigmented melanin containing neurons in the midbrain ^4^. The latter reflects the neurodegeneration of dopaminergic neurons in the substantia nigra (SN) leading to a marked dopamine deficit in the striatum. Since 1961 ^5^, L-Dihydroxyphenylalanine (L-Dopa), a symptomatic dopamine replacement therapy, has been available for PD for more than 50 years. As L-Dopa, the precursor of dopamine—and subsequently ergot- and non-ergot dopamine agonists— are highly effective in reducing motor symptoms, PD was—for a long time—predominantly considered as a movement disorder. This focus was even enforced by the unraveling of the motor circuitry of the basal ganglia, of its imbalances in PD and the dramatic therapeutic effect of deep brain stimulation (DBS) of the subthalamic nucleus or the globus pallidus. These symptomatic therapeutic achievements may explain why the development of therapies for the wide range of disabling non-motor symptoms (NMSs) the patient with PD has throughout the course of the disease has been neglected. In addition, research efforts on the development of disease-modifying drugs were largely performed in acute toxin-induced rodent models (6-hydroxy-dopamine, or MPTP) and their neuroscientific results failed to translate into clinically successful drugs (reviewed in 6). Thus, apart from few cases of toxin-induced Parkinson syndromes, firm knowledge at the molecular level on the etiopathogenesis of PD was lacking until the year 1996.

### Genetic and neuropathological research revolutionize the understanding of Parkinson’s disease

Two discoveries between 1996 and 2006 changed the field:

1) The description of a mutation in the gene for the protein alpha-synuclein, causing a rare form of autosomal-dominant PD ^7^; the discovery that gene duplication of this gene for normal wild-type alpha-synuclein (that is, the presence of three alleles instead of two alleles leading to a production of 150% of normal alpha-synuclein) causes PD; and the presence of pathological alpha-synuclein-aggregations in the Lewy bodies in the SN ^3^.
2) The publication of the Braak staging of PD ^8^ combined with the “dual hit theory” ^9^ (see below) proposing that the manifestation of motor PD symptoms is a late-stage phenotype preceded for years, if not decades, by three prodromal stages ( Figure 2).

### The shift from research on symptomatic therapy to the search for Parkinson’s disease-modifying therapy

Based on these findings, the majority of cases with PD (the so-called idiopathic form of PD) were assumed to present an alpha-synucleinopathy. Drug development shifted its focus from transmitters, transmitter-related receptor agonists and antagonists, and transmitter-synthesizing and -degrading enzymes to the protein chemistry, synthesis, transport, aggregation, and degradation of alpha-synuclein and other proteins related to neurodegenerative disorders, such as MAP-Tau or beta-amyloid. A now-20-year-long effort in neuroscience and drug development appears to provide the first results.

This article summarizes (1) the recent achievements to further improve symptomatic therapy of motor PD symptoms, (2) the still-limited attempts to provide symptomatic therapy for NMSs in PD, and (3) the advances in the development and clinical testing of compounds, which promise to offer disease modification in already-manifest PD. However, prevention (that is, slowing or stopping PD in a prodromal stage) is still a dream and one reason for this is that we have no consensus on primary endpoints for clinical trials, which reflect the progression in prodromal stages of PD, such as in rapid eye movement (REM) sleep behavior disorder (RBD), the most specific prodromal stage of PD - a methodological challenge to be met in the future.

## Present therapy 2017

### Present therapy (2017) available for Parkinson’s disease is symptomatic

In 1960, the lack of dopamine in brains of patients with PD was discovered, and the first rationally derived therapy was introduced to neurology when the “miraculous” improvement of the motor symptoms under therapy with intravenous L-Dopa, the blood-brain barrier-passing precursor of dopamine ^5^, was reported. Since then, generations of medical students have learned that the symptoms of PD are caused by a dopamine deficit, leading to an imbalance of the motor, cognitive, and emotional loops in the basal ganglia circuitry. Although hard to believe, 56 years after its discovery, L-Dopa is still the gold standard for any of the available multiple symptomatic therapies for PD. Owing to L-Dopa’s short plasma half-life (1–2 hours), repeated intake results in a pulsatile plasma profile. With progressing neurodegeneration of the nigrostriatal pathway, the storage capacity of the central nervous system (CNS) for L-Dopa and dopamine declines, thus in the intermediate to advanced stages of PD, the duration of the central L-Dopa effect will mimic the pulsatile plasma profile of the medication. The longer the disease lasts, the more patients with PD experience “motor complications”, consisting of motor fluctuations (a change between phases of akinesia (OFF: no or low, therapeutically ineffective L-Dopa level) and normal movement (ON: therapeutically effective L-Dopa level) and also of an excess of movements (choreatic “dyskinesia”) at the peak of the L-Dopa curve in the blood and thus in the CNS. To delay or ameliorate these L-Dopa therapy-associated motor complications, several other classes of drugs are available to be prescribed before the use of or in combination with L-Dopa. The L-Dopa effect can be enhanced and prolonged by (1) the combination with peripheral inhibitors—(a) of the degrading enzyme L-DOPA-decarboxylase (that is, benserazide, carbidopa – standard combination) and (b) of the degrading enzyme catechol- *O*-methyl transferase (COMT) (that is, by adding the short-acting COMT-inhibitor entacapone or intermediate-acting tolcapone)—or with (2) centrally active inhibitors of the degrading enzyme monoamino-oxidase B (MAO-B), such as selegiline or rasagiline. As an advanced therapeutic option, L-Dopa emulsion can be applied by an external pump via a percutaneous tubing into the jejunal cavity in order to provide a nearly constant continuous supply of L-Dopa to the blood and thus to the CNS. In addition, physicians have at hand five non-ergot dopamine agonists, mainly of the dopamine-2-receptor type: pramipexole, ropinirole and piribedil (only registered in Europe) for oral intake, rotigotine by 24-hour transdermal application, and apomorphine, which needs a parenteral administration (that is, subcutaneous: bolus or pump assisted infusion). As pramipexole and ropinirole are available—besides the standard release formulation—as slow release preparations, four non-ergot agonists can offer continuous dopamine-2-receptor stimulation for a 24-hour period. Ergot dopamine agonists are indicated only as a second-line choice, as they run the risk of inducing fibrosis of the heart valves or the retroperitoneum. Furthermore, the *N*-methyl-D-aspartate (NMDA) (glutamate subtype) receptor antagonist amantadine is considered to improve PD motor symptoms and at the same time to reduce motor complications, especially dyskinesia.

Finally, numerous pharmacotherapies are available for individual NMSs ^10^ though mostly by employing a given compound approved to treat the symptom or disease per se and not a particular symptom as part of the spectrum of NMSs in PD (for example, using an antidepressant for depression in PD; see below and Seppi *et al*. ^11^)

The introduction of DBS further increased the therapeutic options for patients with PD. The pacemaker assisted stimulation of stereotactically, to the millimeter precisely placed electrodes—either in the subthalamic nucleus or the internal part of the globus pallidus—allows reduction of the imbalance in the above-mentioned motor circuitry of the basal ganglia ^12^. This procedure has been shown to be effective not only in very advanced PD patients but also in PD patients, who just have started to develop motor complications ^13^. DBS in turn allows one to decrease the amount of pharmacotherapy and this measure leads to less motor (see above) or neuropsychiatric (see below) adverse effects or both, which can occur with the above-mentioned combination of different pharmaceuticals in all stages of PD.

In addition, in the last decade, an increasing number of partly well-designed and conducted studies have investigated the effect of non-medical/non-surgical supportive “activating” therapies; these include physical exercise, physiotherapy (more than 30 trials) ^14, 15, 82, 83^, dance interventions (4 trials), and logopedic training of dysphagia (2 trials) on the neurological symptoms of PD patients and their quality of life (comprehensively reviewed in 15, 16). Despite all of these achievements until today, we still treat PD at an entirely symptomatic level ^17^.

### The challenge of treating motor AND non-motor symptoms

In summary, neurologists, and especially movement disorder experts, can choose from a large number of compounds to treat the motor symptoms in PD effectively for several years, if not decades. In addition, owing to advances in the field of internal medicine, the surgical disciplines, and anesthesia, patients with PD live longer, but with a price or rather trade off to find the optimal therapeutic balance between motor- and non-motor symptoms with a minimum of adverse effects:

1) With increasing age and duration of PD, gait problems—non-responsive to dopamimetic therapy, combined with the increased risk to fall and to incur fractures—and other NMSs appear. These NMSs include autonomic dysfunctions (for example, urinary incontinence and severe obstipation), sleep impairment, pain syndromes, and most important, neuropsychiatric symptoms, including depression, impulse control disorders, punding, hallucinations, overt psychosis (in part induced by dopamimetic therapy), and cognitive impairment progressing to dementia ^10^. The NMSs substantially impair the quality of life not only of the PD-patient but also of their family. Thus, in the very advanced stages, the family and the physician face—in many cases—a disoriented, often demented PD patient, who either is mobile, if not hypermobile (dyskinetic), and may hallucinate and even endanger him- or herself or others or who is akinetic-rigid.

2) With increasing age, the extent of comorbidity increases (for example, orthopedic syndromes, diabetes mellitus and metabolic syndrome ^16^, heart failure, and stroke). This comorbidity in patients with PD is a major challenge in the ambulatory care. Hardly any studies have been performed to assess the efficacy, benefit-risk ratio, and tolerability of available symptomatic PD therapy in these real-life “wild-type” PD patients.

Thus, the weight of therapeutic need has shifted from “just” making or keeping the PD patient mobile to the challenge to fine-tune a therapeutic combination of drugs for (1) the treatment of motor and non-motor symptoms, (2) motor and non-motor complications (including hallucinations, or psychosis induced by dopamimetic therapy, particularly in cognitively impaired patients with PD), and (3) treatment that, in accordance with other medical treatments and care, is acceptable to the patient and the caring partner.

## New developments in Parkinson’s disease therapy

With this situation in mind, efforts over the last 20 years to develop new therapies for PD can be divided in two categories: (1) improving symptomatic therapy of (1a) motor and (1b) non-motor symptoms and (2) addressing potential causes of PD, with a focus on the protein alpha-synuclein, its chemistry, synthesis, aggregation, degradation, and interaction with other proteins in order to develop a disease modifying treatment.

### New developments in symptomatic therapy of motor symptoms

To improve the available symptomatic therapy for motor symptoms, several drugs have recently been approved or are still under testing. These developments include the improvement of the pump device for infusing L-Dopa in the jejunal cavity (likely available in 2017 or 2018 ^18^) and the approval of a long-acting (5- to 6-hour duration of action) L-Dopa (currently available in the USA under the tradename Rytary ^19– 25^; Table 1). The latter compound is absorbed not only in the duodenum but also in the ileum; thus, its absorption may depend less on food intake/diet ^26^. Surprisingly little information is available about its use in daily practice, and no active comparator trial (for example, against the intrajejunal L-Dopa-infusion or DBS) is under way or in planning in PD patients with motor complications.

**Table 1.** New developments in symptomatic therapy of Parkinson’s disease. **Part A. For motor symptoms and motor complications by means of dopaminergic mode of action: new compound or new formulation, mode of action (fully or in part) provided by other approved compound**.

Compound/ *Trade name* (Company [C], Sponsor [S])	Indication	Mode of action	Phase of development	Commentary and approved dose	Reference
**Melevodopa/** **Carbidopa** *Sirio* (Chiesi [C])	Motor	Modified form of L-Dopa soluble tablet	Approved	Marketed in Italy	Zangaglia *et al*. 2010 ^85^ Fasano *et al*. 2014 ^86^
**Opicapone** *Ongentys* (BIAL [C])	Motor wearing-off	COMT-inhibitor, long-acting, add-on to L-Dopa	Approved	Reimbursed in EU **50 mg/day**	Ferreira *et al*. 2015 ^33^ Ferreira *et al*. 2015 ^87^ Rocca *et al*. 2016 ^35^ Fabbri *et al*. 2016 ^34^
**Safinamide** *Xadago* (Zambon [C])	Motor wearing-off	MAO-B-inhibitor, glutamate modulator, add-on to L-Dopa	Approved	Reimbursed in EU, active comparator study to other MAO-B- inhibitors not available **50 or 100 mg/day**	Stocchi + Torti 2016 ^32^ Cattaneo *et al*. 2016 ^31^ Borgohain *et al*. 2014 ^29^ Borgohain *et al*. 2014 ^30^ Schapira *et al*. 2013 ^28^ Stocchi *et al*. 2012 ^27^
**“XP066”** *Rytary* (Impax [C])	Motor wearing-off	L-Dopa/ Carbidopa (4/1) long-acting, extended release	Approved	Reimbursed in USA **95, 145, 195, or 245 mg** **L-Dopa capsules**	Yao *et al*. 2016 ^25^ Mao + Modi 2016 ^24^ Waters *et al*. 2015 ^22^ Hsu *et al*. 2015 ^23^ Stocchi *et al*. 2014 ^21^ Pahwa *et al*. 2014 ^20^ Hauser *et al*. 2013 ^19^

**Table:** **Part B. For motor symptoms, motor complications or non-motor symptoms by means of a non-dopaminergic mode of action: new compounds or new formulation**.

Compound	Indication	Mode of action	Phase of development	Commentary	Reference
**Amantadine** Extended release (Adamas [C])	Motor dyskinesia off-time	NMDA-receptor antagonist, long-acting	Phase III completed	Likely to be registered 2017 or 2018 as **340 mg/day**	Pahwa *et al*. 2016 ^37^ Pahwa *et al*. 2015 ^36^
**Droxidopa** L-Threo-3,4- Dihydroxy-Phenylserine *Northera* (Lundbeck [C])	Motor and Non-Motor freezing Neurogenic orthostatic hypotension	Noradrenaline precursor		Approved in Japan, approved in USA **3×100 mg/capsule** **max. 3×6 capsules** (max. daily dose 1,800 mg)	Hauser *et al*. 2014 ^56^ Espay *et al*. 2014 ^57^ Mathias *et al*. 2001 ^55^
**Istradefylline** *Nouriast* (Kyowa-Hakko- Kirin [C])	Motor wearing-off	Adenosine 2A receptor antagonist	Phase III positive Phase III ongoing in EU	Approved in Japan **20 mg/once daily** (40 mg/daily possible)	Vorovenci + Antonini 2015 ^43^ Kondo *et al*. 2015 ^40^ Pinna 2014 ^44^ Mizuno *et al*. 2013 ^39^ Pourcher *et al*. 2012 ^42^ Factor *et al*. 2010 ^41^ Mizuno *et al*. 2010 ^38^
**Tozadenant** (Biotie [C])	Motor dyskinesia wearing-off	Adenosine 2A receptor antagonist	Phase III ongoing		Michel *et al*. 2015 ^88^ Hauser *et al*. 2014 ^89^ Perez-Lloret + Morello 2014 ^90^
**Pimavanserin** *Nuplazid* (Acadia [C])	Non-motor psychosis	5HT2A inverse agonist	Phase III positive	Approved in USA **2x17 mg/ once daily**	Cummings *et al*. 2014 ^53^ Hacksell *et al*. 2014 ^91^

Modified from Oertel and Schulz ^17^ (2016).

**Table:** **Part C. For motor complications or non-motor symptoms by means of a non-dopaminergic mode of action: drug approved in another indication, now tested in Parkinson’s disease**.

Compound	Indication	Mode of action	Phase of development	Commentary	Reference
**Donepezil** Eisai (C)	Non-motor falls, gait disorder, dementia in Parkinson’s disease (PD)	Acetylcholin- esterase- inhibitor	Phase IIIb ongoing	Approved for therapy of Alzheimer dementia	Chung *et al*. 2010 ^92^ Ravina *et al*. 2005 ^93^
**Duloxetine** *Cimbalta, Xeristar* (University of Toulouse [S])	Non-motor pain	SSNRI	Phase III ongoing	Approved for therapy of pain and of depression	
**Oxycodone/** **Naloxone** *Targin* (MundiPharma [C])	Severe pain syndrome in PD	Opioid	Phase III positive	Approved for therapy of pain	Trenkwalder *et al*. 2015 ^54^

Modified from Oertel and Schulz ^17^ (2016).

To prolong and enhance the action of L-Dopa, the new reversible MAO B-inhibitor safinamide ^27– 32^—with the claim that this compound also influences glutamatergic transmission—and the long-lasting COMT inhibitor opicapone ^33– 35^ have been approved (both at present in Europe). An extended-release (24-hour long-acting) formulation of amantadine has been recently reported to markedly reduce the severity and extent of L-Dopa-induced dyskinesia and also to reduce off-time ^36, 37^.

Based on the restricted localization of adenosine-2A-receptors in the striatopallidal indirect pathway of the basal ganglia circuitry, adenosine-2A-receptor antagonists have been developed for the therapy of motor fluctuations and are now approved in Japan (istradefylline, tradename Nouriast ^38– 40^, whereas registration trials ^41– 43^ in Europe with this compound and clinical testing with a second compound of this class (tozadenant) are ongoing ( Table 1).

The development of a third compound of this class, preladenant, was stopped because of negative results in phase III trials ^44^.

Likewise employing the primary endpoint of “reduction of L-Dopa induced dyskinesia”, the results of clinical phase III trials with the metabotropic-glutamate-receptor-5-antagonist (mavoglurant) ^45– 48^ and the nicotine-alpha-7-partial agonist AQW051 ^49^ were negative and thus their development stopped, whereas the clinical testing of glutamate-receptor-4-antagonists and allosteric modulators of the metabotropic-glutamate-receptor-5—employing the same endpoint (reduction of dyskinesia)—are ongoing.

For the field of DBS, improved hardware and software (rechargeable battery, multipolar electrodes) now offer a higher flexibility and precision to individually target the different motor symptoms (akinesia, rest tremor) and to avoid stimulation-related side effects (reviewed in 50– 52).

### New developments in symptomatic therapy of non-motor symptoms

In 2011, the International Movement Disorder Society published a comprehensive evidence-based medicine review on the therapy of NMSs of PD ^10, 11^. On one hand, it provides profound guidance for how to treat the individual NMSs in PD. On the other hand, this review demonstrates how few clinical randomized placebo or active comparator multicenter prospective trials of “evidence-based medicine class I and class II” existed thus far for the therapy of NMSs in PD—in contrast to the numerous prospective multicenter trials on motor symptoms and motor complications. Since then, increased efforts in this field have led to the approval of new therapies. For the treatment of dopamimetic-induced psychosis in PD, the 5HT2A inverse agonist pimavanserin has been approved in the USA ^53^; thus, for the first time, an alternative to the currently employed antipsychotic compounds quetiapine or clozapine is available—although an active comparator trial between pimavanserin and, for example, clozapine is missing. In regard to severe pain syndromes, the slow-release preparation of oxycodone/naloxone has been successfully tested in PD ^54^. Furthermore, the precursor of noradrenaline droxidopa, also known as L-threo-DOPS, has been approved for the treatment of neurogenic orthostatic hypotension, one of the troubling autonomic symptoms in advanced PD and even more so in multiple system atrophy (MSA), another alpha-synucleinopathy ^55– 57^. In regard to DBS, a large recent study has shown beneficial effects of this neurosurgical procedure on NMSs ^58^. Given the major impact of NMS and therapy-related non-motor complications on the quality of life for PD patients and their partners, this field clearly needs priority in future clinical trials.

In summary, these compounds and techniques allow fine tuning of the available symptomatic therapy of motor and in part of NMSs in PD. However, they do not represent a major innovation. A true, highly needed innovation would be a compound with disease-modifying properties in order to slow down, if not stop, the progressive pathophysiology of PD (that is, most likely the spreading of the alpha-synucleinopathy in the central, peripheral, autonomic, and gastrointestinal nervous system of patients with PD).

### The revolution in genetic research in Parkinson’s disease

In 1997, the world of PD research changed. For the first time, though very rare, an autosomal dominant mutation (termed *PARK1*) responsible for the protein alpha-synuclein was described ^7^. By 2016, at least eight monogenic causes for PD are known. The autosomal dominant forms relate either to a mutation of alpha-synuclein or to LRRK2, whereas autosomal recessive forms ( *PARK2*, *PINK1*, *DJ1*) cause mitochondrial dysfunction. The third major discovery was the fact that 3–7% of patients with idiopathic PD carry a heterozygous mutation for the gene glucocerebrosidase A. Genome-wide association studies have confirmed—besides the role of alpha-synuclein—the importance of the microtubule-associated protein tau (MAPT) in the etiopathogenesis of PD. Furthermore, at least 28 genetic risk (susceptibility) factors have been identified, and it is likely that this number will further increase (reviewed in 59). These discoveries have already had a major impact on the development of new therapies, especially in regard to potentially disease-modifying compounds—as will be discussed in relation to the alpha-synuclein “spreading hypothesis” below.

### The Braak staging of Parkinson’s disease, the spreading hypothesis, the search for prodromal stages of Parkinson’s disease: advantages and limitations

Shortly after the discovery that a mutation of alpha-synuclein causes PD, alpha-synuclein aggregates were identified in the Lewy bodies in the post-mortem SN samples of patients with idiopathic PD ^3^. Therefore, the majority of patients with idiopathic PD are now considered to suffer from an alpha-synucleinopathy. Based on the distribution of these Lewy bodies in the nervous system, Braak *et al*. ^8^ postulated that most likely PD—as it is defined with its motor symptoms by the neurologist—is a late-stage phenotype of a disease which has been going on for decades.

### The Braak staging of Parkinson’s disease: the spreading hypothesis

The Braak ^8^ staging hypothesis, combined with the “dual hit theory” ^9^, proposes that PD starts either in the olfactory bulb and related areas or in the gastrointestinal system. Thus, a pathological agent either would retrogradely reach the SN via an only recently discovered connection between the olfactory bulb and the SN ^60^ or may move retrogradely from the gastrointestinal system up to the dorsal motor nucleus of the vagal nerve, would then—in a caudorostral ascending direction—propagate upwards in the brainstem reaching the locus coeruleus complex, and over the next 5 to 10 years finally affect the SN (see Figure 2 and its legend).

**Figure 1.**
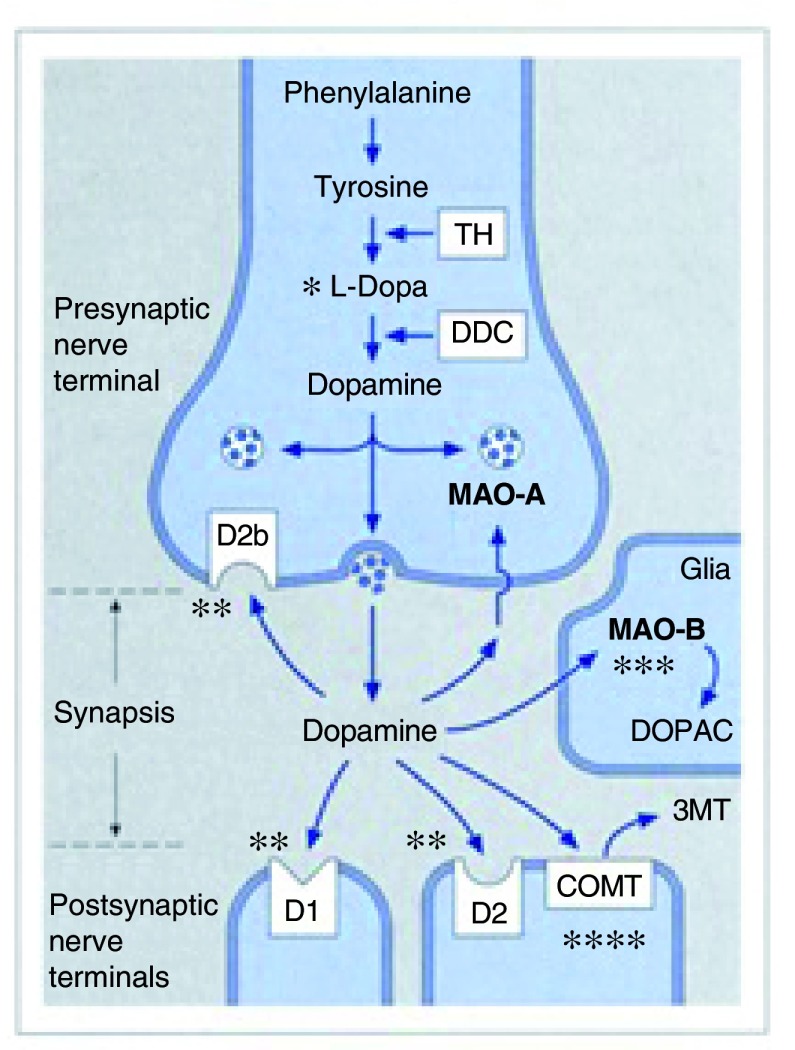
Scheme of a dopaminergic synapse with the different sites of actions for symptomatic Parkinson’s disease therapy. COMT, catechol-O-methyl-transferase (inhibitor: entacapone, opicapone ^a^, tolcapone); D1, D1 receptor: agonist (new compound in phase III); D2, D2/D3 receptor (non-ergot agonist: apomorphine, piribedil, pramipexole, ropinirole, rotigotine); D2b, presynaptic D2-autoreceptor; DDC, L-DOPA-decarboxylase; DOPAC, dihydroxyphenylacetic acid; L-DOPA ^a^, L-dihydroxyphenylalanine (extended release); MAO-A, monoaminooxidase A; MAO-B, monoaminooxidase B (inhibitor: rasagiline, safinamide ^a^, selegiline); TH, tyrosine hydroxylase; 3MT, 3 methoxy-tyramine; *, effect of L-DOPA (converted into dopamine); **, effect of dopamine agonists (mimics dopamine at D1 or D2/3 receptor and at D2b autoreceptor); ***, effect of MAO-B-inhibitor (blocks centrally degradation of dopamine, enhances and prolongs dopamine action); ****, effect of COMT-inhibitor (blocks peripherally degradation of L-DOPA, enhances and prolongs presence of L-DOPA in blood and thus enhances and prolongs action of central dopamine action); 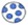, vesicle; the round structure represents the symbol for the vesicle 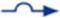, the arrow represents the uptake mechanism - dopamine reuptake mechanism. ^a^Recently approved for therapy of Parkinson’s disease ( Table 1).

**Figure 2.**
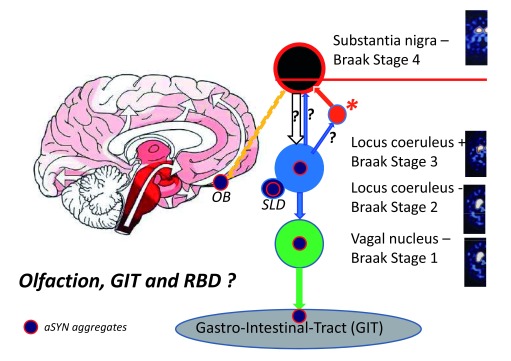
Braak’s hypothesis: staging of Parkinson’s disease. This simplified hypothetical scheme shows how Parkinson’s disease may develop based on the hypothesis of the Braak staging ^8^ and the “dual hit theory” ^9^. OB, olfactory bulb, SLD, sublaterodorsal nucleus of the subcoeruleus/coeruleus complex; lesion of the SLD leads to rapid-eye-movement sleep behavior disorder (RBD). The orange line illustrates the afferent connection from the substantia nigra to the olfactory bulb. The α-synuclein (aSYN) aggregates (red-circled icon) may retrogradely spread from the GIT via the dorsal motor nucleus of the vagal nerve to the locus coeruleus and subsequently ascend to the substantia nigra (Hit 1). It is discussed that the later propagation may either take place by means of direct anterograde transport (thin light blue arrow with question mark – from the LC to the SN) or retrograde transport from the LC to the SN (empty arrow with question mark), or via an intermediate structure (orange circle) such as the amygdala complex (asterix). Likewise the aSYN aggregates (red-circled icon) may retrogradely spread from the OB to the substantia nigra (Hit 2). Dopamine transporter single-photon emission computed tomography (DAT-SPECT): On the right side, images of the striatal presynaptic terminals of the nigrostriatal pathway are visualized by DAT-SPECT. In Braak stage 1, the DAT-SPECT is normal. In Braak stage 2, the DAT-SPECT is still normal (Locus coeruleus −). In Braak stage 3, the nigrostriatal pathway is partially degenerated but less than necessary to cause parkinsonian motor symptoms and signs. The image shows a partial loss of the nigrostriatal dopaminergic terminals in the dorsolateral aspect of the striatum (Locus coeruleus +). The red line indicates the threshold for the extent of the nigrostriatal lesion associated with parkinsonian motor symptoms. In Braak stage 4, the loss of the nigrostriatal terminals in the striatum is so marked that the patient will experience parkinsonian motor symptoms and signs.

There is considerable clinical and experimental evidence in favor of this “dual hit theory” ^9^, although it is not proven beyond doubt and likely not to be applicable for a minority of patients with PD. That alpha-synuclein can spread in the human nervous system was reported in 2008 ^61^: Lewy bodies arise in healthy fetal mesencephalic cells several years after their transplantation into the striatum of patients with PD. This fact is most likely explained by trans-synaptic spreading of pathological alpha-synuclein from the host neurons into the donor neurons. Intense research in experimental animal models of PD is ongoing to understand the pathogenic role of the different forms (such as monomers versus oligomers versus fibrils) of aggregated alpha-synuclein and to analyze the mechanism behind the postulated “prion-like” spreading of alpha-synuclein. For example, research in a transgenic alpha-synuclein-overexpressing and germ-free mouse model of PD recently demonstrated a marked effect of the gut microbiota on the development of the alpha-synucleinopathy and the manifestation of motor impairment, thus strengthening the hypothesis that the gastrointestinal system can play a role in the etiopathogenesis or progression (or both) of PD ^62^.

### Clinical research on prodromal Parkinson’s disease

In the clinical situation, manifest PD—according to Braak *et al*. ^8^—is preceded by years, if not decades, by prodromal phases. To screen for prodromal (premotor) phases, the NMS hyposmia, constipation, depression, and the sleep-dream phase disorder RBD (REM sleep behavior disorder) are now considered prodromal indicators. Whereas the first three are sensitive but not specific, RBD is now accepted as the most specific phenotype of the PD prodromal phases with a risk of more than 80% to convert into PD, or dementia with Lewy bodies (DLB) or less frequently into multiple system atrophy(MSA)—in 10 to 15 years ^63, 64^. Similar research on prodromal stages takes place with “at risk relatives” of Parkinson patients, who are either heterozygous for the *LRRK2* gene or are homozygous for one of the autosomal-recessive genes for a mitochondrial dysfunction in PD ( *PAKR2*, *PINK1*, *DJ1*; for a comprehensive review on Mendelian and non-Mendelian inheritance of PD, see 59) or possess a mutation of the gene for glucocerebrosidase 1 ( *GBA1*) and thus are classified as PD-GBA1 ^65^.

### The Braak hypothesis: advantages and limitations

The advantage of the Braak hypothesis is that it can clinically be tested. By carefully screening and following up patients at risk for PD, such as patients with hyposmia or RBD or with both ^66^, clinicians can identify a subgroup of patients with prodromal PD who present with and develop the sequence of symptoms related to the postulated prodromal PD stages. This type of study may allow us to discover endpoints for future neuroprotective trials in prodromal PD.

Limitations of the Braak hypothesis are related to post-mortem analysis of PD brains ^67^: they show, for example, that the density of Lewy bodies in the medullary areas is lower than in the cortex. In addition, a similar distribution of Lewy neuropathology is observed in patients with incidental Lewy body disease (that is, individuals with the hallmark Lewy pathology in brain, who did not present when alive with motor Parkinson features).

This observation does not appear to be consistent with a caudorostral spreading of alpha-synuclein aggregates. But if Lewy bodies are considered a mechanism to reduce the amount of soluble toxic alpha-synuclein oligomers in the cell, then the density of Lewy bodies in a given brain area may reflect its “defense” capability. In addition, if the caudorostral ascending process is tightly linked to the connectome of the involved structure, the locus coeruleus—with its lack of connections to the basal ganglia and, on the other hand, its strong projections to cortical areas—might drive the alpha-synuclein load of cortical areas many years longer than the SN might influence the alpha-synuclein load of the basal ganglia. This speculation is again testable in animal models and in post-mortem studies.

### Search for Parkinson’s disease-modifying therapy delivers first results

The ultimate therapeutic challenge remains. Every person who knows a patient with PD asks why there is not a treatment that prevents the (at least) motor manifestation of PD. Already-diagnosed PD patients and their families dream of a treatment that can slow down or even partially reverse the progression of this devastating disorder with all its motor and non-motor symptoms and complications in the advanced stage. These dreams may be fulfilled in the not-too-distant future.

Two therapeutic strategies are currently followed. The first is based on epidemiological findings and large clinical prospective trials reporting a correlation between a reduced occurrence or prevalence (or both) of PD and the consumption of compounds such as caffeine or nicotine (e.g., 68). Table 2 lists examples of these generic substances with a postulated disease-modifying potential for PD.

**Table 2.** Therapy with compounds of disease-modifying potential: generic substances.

Compound	Indication	Mode of action	Phase of development	Reference
Caffeine (University of Montreal, Canada [C])	Motor early Parkinson’s disease (PD)	Adenosine-receptor antagonist	Phase IIIb ongoing	Wills *et al*. 2013 ^94^ Postuma *et al*. 2012 ^95^
Inosine (Michael J. Fox Foundation [MJFF] [C])	Motor early PD	Precursor of urate, antioxidant	Phase IIb ongoing	Bhattacharyya *et al*. 2016 ^96^ Ascherio *et al*. 2009 ^97^]
Isradipine – STEADY-PDIII (NIH-NINDS, Novartis, University of Chicago [C])	Motor early PD	Dihydropyridine calcium channel blocker	Phase IIIb ongoing	Simuni *et al*. 2016 ^98^ Simuni *et al*. 2013 ^99^
Nicotine - NIC-PD (German Parkinson Study Group, Parkinson Study Group USA; MJFF, IPF, NP, DPG, Novartis Germany [C])	Motor *de novo* PD	Cholinergic, modulation of α-synuclein aggregation?	Phase IIIb completed	Oertel *et al*. 2016 ^100^ Quik *et al*. 2008 ^101^ Hong *et al*. 2009 ^102^

Modified from Oertel and Schulz ^17^ (2016).

The second approach relates to the groundbreaking genetic discoveries in PD. In fact, a dramatic shift in the strategy for developing a new PD therapy has taken place: pharmaceutical efforts now target alpha-synuclein protein synthesis, degradation (such as autophagia, lysosomal, or proteasomal degradation), protein aggregation, and propagation in the nervous system. Finally, 20 years after the discovery of *PARK1*, the academic and pharmaceutical industrial scientific community can offer the first candidates with a potential for a disease-modifying effect in PD.

Three different principles of therapeutic action are addressed: (1) active or passive immunotherapy, (2) modulation of alpha-synuclein aggregation, and (3) enhancement of autophagy of alpha-synuclein ( Table 3).

**Table 3.** Therapy with compounds targeting alpha-synuclein.

Compound	Indication	Mode of action	Phase of development	Reference
*Immunotherapy (IT)*				
Active immunization (Affiris [C])	Motor	IT	Phase II ongoing	Schneeberger *et al*. 2016 ^103^ Manoutcharian *et al*. 2016 ^104^
Passive immunization BIIB054 (Biogen [C]) PRX002 (Parthena/Roche [C])	Motor	IT	Phase II in preparation phase II in preparation	Weihofen *et al*. 2016 ^105^ Bergström *et al*. 2016 ^106^ Kalia *et al*. 2015 ^6^ Games *et al*. 2014 ^107^ Spencer *et al*. 2016 ^69^
*Alpha-synuclein* *aggregation modulators* *(aSAMs)*				
NPT200-11 (UCB/Neuropore [C])	Motor? likely in *de novo* Parkinson’s disease (PD)	aSAM	Phase I in planning	Koike *et al*. 2014 ^74^ Szoke *et al*. 2014 ^75^
*NPT100_18a* (Neuropore [C])	Not applicable	aSAM	Preclinical testing	Wrasidlo *et al*. 2016 ^75^
ANLE 138b (MODAG [C])	Motor? likely in *de novo* PD	aSAM	Phase I in planning	Deeg *et al*. 2015 ^73^ Levin *et al*. 2014 ^72^ Wagner *et al*. 2013 ^71^
*a-synuclein autophagia* *enhancer (aSAE)*				
Nilotinib *Tasigna off-label use* (Georgetown University, Washington, DC, USA [C])	Motor non-motor	“Tyrosine kinase inhibitor” aSAE	Investigator initiated trial – open-label small pilot study randomized controlled trial in planning (MJFF-USA, Cure PD Trust, UK)	Pagan *et al*. 2016 ^78^ Hebron *et al*. 2014 ^80^ Hebron *et al*. 2013 ^81^

Modified from Oertel and Schulz ^16^ (2016).

1) The first approach mimics a strategy that has been followed in Alzheimer’s disease for the last decade: active and passive immunizations are being developed as therapeutic approaches. This immunotherapeutic strategy relies on the assumption that (a) alpha-synuclein is accessible in the extracellular space (trans-synaptic spreading), (b) antibodies against alpha-synuclein reach the brain in sufficient quantity, and (c) they trap alpha-synuclein aggregates when these are released (“spread”) into the extracellular synaptic space. Today, active and passive immunization trials are under way in phases I and II. These treatments have passed the safety level testing, and the first data on phase II trials are awaited in 2017 to 2019. One limitation of active and passive immunotherapy, the low amount of antibodies passing the blood-brain barrier, may be overcome by coupling antibodies to the peptide penetratin, as has recently been reported in a mouse PD model ^69^.

2) Modulating the aggregation of alpha-synuclein aims to block or reduce the aggregation of alpha-synuclein monomers to oligomers or later on to fibrils. Two drugs are close to or under very early development. The first compound is called ANLE138b, and the few articles published demonstrate that this drug is able to reduce the aggregation of alpha-synuclein. In addition, in a mouse model with an A30P alpha-synuclein mutation, the compound extends survival ^70– 72^. The second drug is called NPT200-11, and only abstracts on its efficacy in preclinical testing are in the public domain ^73, 74^. This compound again reduces aggregation of alpha-synuclein at least *in vitro* and according to public information should have reached the very first safety testing in humans. A third compound NPT100-18a has been reported to displace alpha-synuclein from membranes, but is still in the phase of preclinical testing ^75^. The advantage of these small molecules is that, in variance to antibodies employed in immunotherapeutic attempts, they readily pass the blood-brain barrier.

3) Other newly developed compounds promise to enhance autophagy of alpha-synuclein. They are still in preclinical testing, although screening of libraries of registered compounds may well reveal further potential members of this group ^76^.

In 2015, a report from a research group at Georgetown University (Washington, DC, USA) may unexpectedly reduce the time to an available registered and reimbursed disease-modifying therapy of PD ^77^. The neurologists treated 12 patients with PD in advanced stages—including PD patients with cognitive impairment—with the compound nilotinib, a tyrosine kinase inhibitor approved as a therapy for chronic myeloid leukemia. Patients with PD were treated in a safety study with an open-label design (150 or 300 mg daily), and after 6 months of therapy, a clinical improvement was reported. In articles published between 2012 and 2014, nilotinib had been shown to reduce alpha-synuclein levels in protein aggregation models of PD in rodents and to prevent the loss of dopaminergic neurons in a transgenic PD mouse model. Its mode of action is to increase autophagy ^78, 79^. In the meantime, the Michael J. Fox Foundation (MJFF), together with Cure Parkinson’s disease trust in the UK, picked up this finding, which initially was looked at very skeptically, and decided to test nilotinib in a double-blind controlled study in patients with PD.

In summary, the field has steadily shifted from developments on symptomatic therapy to preventive therapy, with at least five different options (active immunization, passive immunization, two small molecules that function as alpha-synuclein aggregation modulators, and most recently an autophagy enhancer with a known adverse profile, which is already registered in the field of oncology). Thus, for the very first time, the possibility of a disease-modifying therapy appears to be testable in PD.

### Search for primary endpoints reflecting the progression of Parkinson’s disease in the prodromal stages

Taking together the discoveries on the genetic background of PD and the Braak staging hypothesis, new avenues for drug development and clinical testing have opened up. For clinical testing—at least in the next few years—potential disease-modifying compounds are and will be tested in the early stage of motor PD; that is, very early *de novo* PD patients, who never received a symptomatic therapy will be recruited and should present with a unilateral asymmetric very mild motor symptomatology.

However, for “true” neuroprevention (that is, the prevention or delay of the conversion of a prodromal stage to the motor stage of PD), parameters and biomarkers which reflect the progression of the alpha-synucleinopathy in the prodromal stage have to be discovered. In addition, such a parameter must be responsive to therapy, even in the prodromal stage, in order to qualify as a primary endpoint for pivotal registration trials. At present, such a parameter has not been identified. Respective research ranges from studies on biomarkers in the cerebrospinal fluid, peripheral blood, saliva, and sweat and in biopsies of the colonic enteric nervous system, the salivary gland, or the skin ^80– 83^. Major efforts are placed into different imaging techniques with sophisticated magnetic resonance methods, nuclear medical ligands for the dopamine transporter single-positron emission computed tomography (SPECT) or fluoro-desoxyglucose positron emission tomography ^84^.

## Conclusions

Neurologists have to accept that the majority of patients with PD, even at the very early stage of neurological diagnosis, actually present a late-stage phenotype of an alpha-synucleinopathy. Thus, PD has started at least 20 years before it manifests in the clinic with its motor symptoms. Neurologists will likely have to shift their clinical and diagnostic focus away from the dopaminergic system to symptoms related to different parts of the nervous system, such as the enteric system ^62^, the brainstem with its autonomic control areas, the locus coeruleus ^57^, or even the skin. If the dream of a disease-modifying therapy is to come true, neuroscience, drug development, and physician scientists face at least two challenges. First, drug development will target the aggregation and propagation of alpha-synuclein and of related mechanisms as well as mitochondrial dysfunction; second, a major effort has to be made to enhance the diagnostic methodology in order to identify a primary endpoint for clinical neuroprotective trials, not only in early motor PD but also in the prodromal stages of PD ^82– 84^. It has never been so exciting as today to work in the field of PD, and we should share this belief with the patients we diagnose, treat, and care for.
